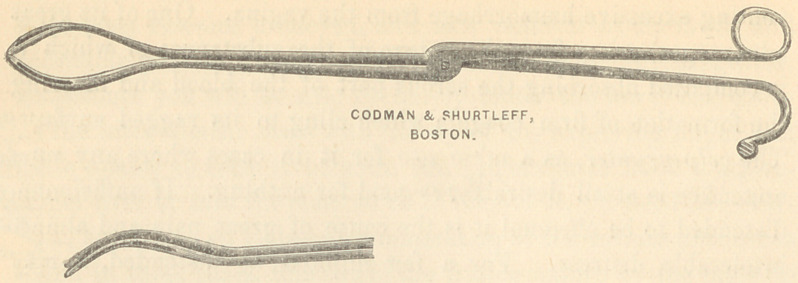# Of Miscarriage, and Especially of the Use of Loomis’ Forceps in Its Management

**Published:** 1879-06

**Authors:** Henry A. Martin

**Affiliations:** Boston, Mass.


### Article III.

### Of Miscarriage, and especially of the Use of Loomis' Forceps in its Management. By Henry A. Martin, M.D., of Boston, Mass. Read before the Norfolk Medical Society, Dec. 11th, 1877.

It is not my intention to offer a paper on the whole very important subject of *abortion*, but merely a few remarks on the treatment of a class of cases, of very frequent occurrence, and very often of a very tedious and troublesome character. That

I should make these remarks was the suggestion of one of our younger members, who was very much struck by the facility with which the contents of the uterus were removed by the use of an instrument not so generally known and appreciated as it should be. What was novel and interesting to him he thought might be also new and interesting to others, and hence his suggestion.

*Honest* miscarriage is a very frequent occurrence among the lower laboring classes, as a result of hard work and all the various shocks which that sort of flesh is heir to. *Dis*honest miscarriage is a very frequent occurrence in the upper and so-called better classes, principally as a result of criminal proceedings with the special object of inducing it. In thirty-three years of practice I have treated a very large number of cases of unpremeditated abortion, a certain number in which I ascertained it to be intentionally induced, and a good many in which I was informed that it was accidental, but in which I felt very confident that it was not so. I do not propose at all to discourse upon the moral aspect of abortion and have felt very little inclined that way since hearing, many years since, a very moral and highly Pecksniffian oration on the subject from one of our brethren, who to my certain and absolute knowledge, had added largely to his income by the practice of this infernal specialty.

Whether accidentally or intentionally induced, there is very little difference in the character and difficulties which abortion presents to the practitioner. However produced, there is one thing essential in the treatment of these cases, viz., to empty the uterus as soon as that can be done with safety, to thoroughly ascertain that it is entirely emptied and never to cease supervision of the case until that end is accomplished. So long as a particle of the ovum remains in the uterus there is danger, great and imminent danger of exhausting and most permanently injurious, if not fatal, hæmorrhage, a lesser and less imminent danger of septic absorption and its miserable train of consequences. The books tell you that when called to a case of threatened miscarriage, with intermitting contractions of uterus, discharge of coagula, pain in the back, etc., you are to give opium, prescribe the recumbent position and take various other steps, all with a view of arresting abortion and saving fœtal life. I have repeatedly followed these time-honored directions ; in some instances I have succeeded in postponing the completion of abortion for weeks, even months, but never that I can remember in preventing its final accomplishment. That in some cases of threatened abortion it is worth while to pursue treatment in the hope of averting it I will not deny, but in a very large proportion of these cases such treatment is bad, serving only to protract the duration of the case and aggravate the injury to the health of the mother. In certainly ninety per cent. of all the cases I have seen, the thing clearly to be done, and as soon as possible, without violence and undue manipulation, has been to empty the uterus.

How is this to be done? In some few cases very easily ; cases in which the os uteri is sufficiently dilated and the ovum or its remains sufficiently protruded and accessible. In such cases, the patient lying on the left side, in the usual position, the left hand of the physician applied with moderate pressure to the lower part of the abdomen over the fundus, the two first fingers of the right hand introduced into the vagina seize the protruded portion of the ovum between them, and by gentle, steady, gradual traction all may be removed *en masse*. Simple as is this little operation, I have known a case in which a woman came very near to death for want of it. A practitioner was called to a woman with the usual symptoms of abortion, was told by the attendant beldame that "it" had all "come away." He took her word for it and visited the patient daily with laudable assiduity, doubtless not forgetting each day to charge his visit. Every day and all day she bled ; he gave opium, sugar of lead, brandy, beef tea and all the rest of it, but never made an examination *per vaginam*, because the old woman had told him that all had "come away," although indeed nothing had been discharged but the fœtus ; a very frequent mistake and one against which the physician should always be on guard. On the evening of the ninth day of his attendance he informed the husband that although he had done so much, had done everything, the woman would almost surely die within twenty-four hours.

Under these circumstances I was called in, removed with my fingers and with the utmost ease, a mass of coagulum and *debris*

of the ovum of the size of a large English walnut, in a putrescent state, which was lying just within the os uteri. A full dose of ergot was given, bleeding stopped at once and, after a few days of doubt, the woman rapidly recovered. I was quite unaware, when I made the visit, that any physician had been in attendance and rated the husband soundly for his neglect only to be told that an experienced one had been in daily attendance for nine days. The doctor was, I believe, very indignant that I stepped in to invalidate his prognosis, and I suppose, if I had known of his attendance, which I am very glad I did not, that medical etiquette would have demanded this in addition to an army of previous similar victims. This case illustrates the absurdity of depending on the statement of anybody in regard to the evacuation of the uterus in these cases. I always ask to *see* what has been discharged, examine this completely. If I find what satisfies me *perfectly* that the ovum has been *entirely* expelled, I save myself and the patient the trouble of an examination ; but I do not find this in one case in twenty. The fœtus, almost always, except in very early abortions, comes away separately and alone ; this is one of the earliest, sometimes *the* earliest symptom. It is a very common notion with even the most erudite of the matrons who infest these cases ; ladies who repel with great scorn and feeling the slightest imputation on the accuracy and fullness of their knowledge and experience, that this is all that is to come. Sometimes a number of coagula are expelled ; some of these often present an appearance of and, on examination, even a pseudo-membranous texture in parts which may deceive the inexperienced practitioner and lead him to suppose that the total membrane and elementary placenta is present, but if carefully broken up in a basin of water it becomes evident that they are but masses of coagulated blood. Such masses in the uterus and protruding through the os are sometimes mistaken for the ovum, are removed and attendance discontinued, when in fact nothing has really been accomplished for the patient's relief.

In some cases you will find the membranes protruding from the os so that they may be seized between the fingers or the jaws of a polypus or other similar forceps, or by some such instrument as Dewees' placental hook ; but the part seized breaking away, the main mass remains unmoved, and this removal of its lower and accessible portion renders the case much more unmanageable. Sometimes you will be able to barely touch the lower extremity of the intra-uterine mass with the tip of the exploring finger, but quite unable to seize it between the fingers or to hook the fore-finger over or into the mass so as to remove it. I need not, however, enter into an elaborate enumeration of the various difficulties found in these cases, due to (1) the friable nature of the mass to be removed; (2) an undilated condition of the os and cervix uteri; (3) the adherence of a portion of the mass to the uterine wall. They are familiar to all who have had any considerable experience in such cases and may be imagined by those who have not. To the theoretical student or practitioner it may seem a very simple and easy matter to take almost any sort of crotchet or hook, like that devised by Dewees, or any sort of a polypus or uterine forceps and remove the aborted ovum. Practically, however, it is by no means an easy matter. It is very easy generally to remove the lower portion of the mass, but doing this is doing nothing, less than nothing. So long as any portion remains in the uterus, any portion however small, attached to the uterine wall, the dangers of the case still remain. The books tell you to wait, to thrust a sponge tent into the os sometimes, to tampon the vagina; the first with a view to dilate the os and so enable the operator to reach and remove the ovum, and the second to prevent injury or even fatal loss of blood from the often very profuse hæmorrhage.

Lately an instrument with the fascinating name of *Colpeurynter*, has been invented by a Dr. Braun, and is much admired by that modern school of physicians (so much lauded by our precious *Boston Medical Journal*), who are ready to applaud any gim-crack so it be invented in Germany, and by those who have never attended a single bad case of hæmorrhage from abortion. It is a bag of thin smooth rubber; introduced into the vagina in a flaccid empty state, it is then distended with air by means of a sort of bellows, and is intended as a substitute for the old fashioned tampon composed of soft old rags, raw cotton, sponges, or similar substances. The tampon is bad enough, but if properly introduced, which it generally is not, it is pretty effectual in preventing excessive hæmorrhage from the vagina. One of its great advantages, arises from the nature of the substances of which it is composed absorbing the serous part of the blood and favoring the formation of firm coagula which cling to its ragged surface. The *colpeurynter*, as a substitute for it in cases where any such appliance is at all desirable, is good for nothing. If sufficiently distended to be effectual it is the cause of great pain and almost intolerable distress. For a few minutes, so distended, it may prevent the flow of blood past it, but does so only for a few minutes, after which the fluid blood finds a free passage between its smooth surface and the expanded and expanding wall.

When the first *colpeurynter* was brought to Boston it was exhibited to an admiring company of physicians. I alone objected to it, theoretically, and since that time my objection to it, at any rate as a substitute for the tampon, has been amply sustained by practical observation and experience. It is worthless, worse than worthless, for the inexperienced physician, having introduced it, relies on it while his patient is bleeding excessively, perhaps to death or very near it.

That it may be and is of use in some of the requirements of gynæcology, I am well aware. My objections to it are as a substitute for the tampon as a means of preventing excessive uterine hæmorrhage. It is not, however, necessary for me to further discuss this very over-estimated novelty. In the vast proportion of cases in which either the tampon or *colpeurynter* is employed neither is necessary or advisable. The cases may be easily and safely terminated, so far as removal of the ovum and consequent cessation of hæmorrhage is concerned, by the proper and skilful use of the very ingenious instrument called *Loomis' forceps.*

I am quite unable to give you any account of the time and place of its invention and it is needless for me to describe it very elaborately, as I offer it for your examination.

[Through the courteous loan, from Messrs. Codman & Shurtleff, of a *cliche* from one of their wood-cuts, I am enabled to introduce here excellent illustrations of Loomis' forceps. (1.) With the blades as when the ovum is embraced between them ; (2), the blades, one within the other, as they should be when introduced into the uterus.]

I purchased this instrument more than ten years ago. I have used it in a great many, probably hundreds of cases, and since I became quite familiar with the proper method of using it, with *invariable* satisfaction and success. I strongly commend it to your consideration and adoption. You will perceive that it consists essentially of two miniature curved fenestrated blades like those of an ordinary obstetric forceps, attached to shanks, at the extremity of one of which is a ring like that of the proximal end of a scissor blade, while at the extremity of the other is a sort of hook or crotchet which fits over the outside of the ring. The entire length of the instrument is about 14 inches. Midway in this length the two shanks are united by a very ingenious mechanical device, a joint, the technical name of which I am ignorant of, but by means of which the blade connected with the hooked handle may be made to revolve to a certain limited extent. It is not very easy to describe the instrument intelligibly but you see at once, on examination, the peculiar mechanism and its object. When the hooked handle is applied to the outside of the ring the two curved rings lie closely one within the other, just like two spoons laid together. With the two blades so laid one within the other the instrument is ready for use. The patient is to be laid on the left side, with the breech at the very edge of the bed, the trunk at an angle of 45 degrees, with its side and the knees well drawn up towards the chest. The tip of the forefinger of the left hand of the operator is to be applied to the posterior side of the *os uteri*. The instrument, properly warmed and well smeared with lard or cosmoline or vaseline, either of the latter the best possible substance for such purposes, held in the right hand, is to be introduced. The convex surface of the conjoined blades gliding over the left forefinger to the *os* are to be gently passed into the uterine cavity with care, that they be made to sweep over its posterior wall. Unless the *os* is very unusually rigid and undilatable this can almost always be done very easily. Even where there is considerable rigidity and deficient dilatation, a little gentle manipulation will enable the blades to pass beyond the cervix. In some cases it may be necessary to introduce a sponge tent to sufficiently dilate the cervix to admit the instrument. This is something, however, that I am very much disinclined to do, feeling sure that the introduction of tents of compressed sponge is by no means so universally innocuous a proceeding as seems to be thought by many practitioners. I have found it necessary to dilate the *os* by sponge tents in but two cases of miscarriage. Generally a few minutes gentle dilatation with the forefinger will be sufficient. I need hardly say that all manipulation, whether with the finger or the instrument, should be gentle, as indeed in all obstetric operations. There can be no doubt that obstetric operators are sometimes oblivious of those two precious axioms *festina lente* and *arte non vi*, but although some of the operative proceedings in this branch of surgery require very considerable muscular strength, it should be strength tempered by gentleness, and this I have more than once witnessed *not* to be the opinion, or at any rate *the practice*, of men who, deservedly or not, have stood very high in the obstetric part of our profession. It is very important that the convex surface of the conjoined blades should be pressed against and glide over the posterior wall of the uterus. If this is not carefully attended to the point of the instrument is pressed against the lower extremity of the mass to be removed, the operator thinks that it has reached the *fundus*, takes the next step of the operation, and withdrawing the instrument, necessarily withdraws nothing with it, and accomplishes nothing. If the instrument is properly introduced its extremity reaches the fundus with great ease. When this is accomplished in an abortion at the most usual time, say fourteen weeks, it will be found that the entire fenestrated portion and from one inch to even two inches more of the instrument has passed beyond the *os*. When the extremity of the blades has reached the *fundus*, to do which, in some cases a portion of the ovum is separated from the uterine wall, the next step is to detach the crotchet from the ring handle to the right, slowly turning it in that direction till it has described half of a circle. When this is done, supposing the blades to have been fully introduced to the fundus, the contents of the uterus are necessarily included between them. The handles should next be made to revolve together once, with a view to fully detaching the *ovum* from the uterine wall. If the *cervix* is sufficiently dilated there is nothing more to do but to withdraw the instrument, which will be found to bring away the *ovum* or all of it that may have remained in the uterus. In many cases, however, the expanded blades cannot be withdrawn. In such cases the forefinger or two first fingers of the left hand must firmly support the uterus while gentle traction with the movement which the French call "a bascule," should be made with a view to dilate the *cervix*. In many cases a very slight amount of dilating force applied from within, in the end, will accomplish sufficient dilatation. In others, five or ten of patient, gentle dilatation may be necessary before the blades can be easily and safely withdrawn. In some cases, a small proportion of all, it may not be possible to withraw the expanded blades without an amount of manipulation hardly to be advised. In these cases it is well to withdraw the instrument and to re-introduce it with the blades applied to each other not so closely as before directed, but so that when introduced to the ovum they may be opened like the mandibles of a duck, pushed up towards the *fundus*, one blade gliding over the posterior, the other over the anterior wall of the uterus, with the intention of including the ovum between them. When this has been done. the operator brings the blades together as when the instrument was first introduced, and when it is withdrawn it will generally be found to bring the ovum or its *debris* with it. It is not easy for an unpracticed writer to describe very intelligently the simplest operation of surgery, and I can hardly hope to have succeeded where so many fail, but nothing can be simpler or more effective than this operation, if its different steps are deliberately and properly taken. The position of the patient is very important to a proper, easy and effectual accomplishment of its object. I know no instrument devised for the very important end of removing the *ovum* in abortion at all comparable in efficacy with this. With the smaller bladed instrument* there are very few cases of miscarriage in which the great end of safe and rapid detachment and removal of the *ovum* cannot be accomplished. To gentlemen who have had no practical experience, sundry devices of the instrument-makers seem very charming, which, in practice, are found to be worse than worthless, and I know no other instrument with which it will not be found very difficult indeed, often impossible, to accomplish the *perfect* removal of the *ovum*. The object of this paper was chiefly to describe the use of *Loomis' Forceps*, and I have little more to say except a few words on a class of cases of abortion, very likely to deceive the practitioner if he depends on a partial removal of the ovum and trusts to luck that the rest will take care of itself. In a certain proportion of cases the ovum is very firmly attached to or near the fundus. The practitioner may pick away with his fingers or some hook or forceps, a large part of it; he may now think that he has got all away, for by an ordinary examination his forefinger discovers nothing remaining in the uterine cavity, and still he has really accomplished nothing, for at the *fundus* remains firmly attached, that portion of the chorion in which has commenced, or if abortion had not occurred would have soon been developed the formation of the placated mass. So long as a particle of this remains, there is no safety from hæmorrhage. I have known bleeding to go on for weeks, for months, when its only and unsuspected cause was a portion of the membrane of the ovum not larger than a cent, attached to the *fundus*. If a second physician had not been called in and by prompt and decided measures removed this placental atom, the patient would most surely have died, as it was she barely escaped. I have known a case of miscarriage at the end of the fifth month, in which a midwife forcibly extracted the placenta, or thought she had done so. The woman got up in a few days, and went about her usual avocations ; five weeks after getting up, while standing in her kitchen, all at once a large black mass fell from vagina to the floor, and she began to flow very freely. She fainted, and I was sent for. I found on the

* The instrument which I have always employed is of the sort which was made at first with unnecessarily large blades. A smaller and lighter instrument is now made and is to be preferred. One of these was exhibited to the society when the paper was read.

floor an immense coagulum exactly moulded to the cavity of the distended uterus, and a profuse hæmorrhage going on. I passed my hand into the vagina, found the os well dilated, and after cleaning out much coagulated blood from the cavity of the uterus, discovered firmly attached, a little to one side of the *fundus*, a mass of the size of a medium English walnut, which, with some difficulty, I detached and took away. It was a piece of the placenta which the midwife had failed to remove six weeks before. It was firmly attached to the uterine wall, was not at all decomposed, and indeed seemed attached to the uterus in such a way as to be nourished by the vascular supply of the uterus. I could narrate, from my own experience, a great many illustrations of the great importance of removing every particle of the *ovum* in cases of abortion, but it is needless. I have no doubt that you all fully appreciate the importance of doing so, and my intention was not a discourse on the whole subject, but rather on a part, a very important part, of the treatment of these accidents.

I cannot resist, however, availing myself of the opportunity to narrate *one* experience of mine, particularly as I much desire to know whether any of my professional brethren of this society have ever encountered anything at all like it. A good many years ago I was called to a woman at the end of the fourth month of pregnancy. She was flooding, had intermitting pains, in a word, was miscarrying. She was well advanced in life, considerably over 35 years of age, and this was her first pregnancy. As is not unusual in similar circumstances, the *os uteri* and *cervix* were rigid and undilatable and I had a tedious time of it, being three days in attendance before the entire ovum was removed. When my attendance ceased, I impressed upon her the importance, in case of a future pregnancy, of her notifying her physician as soon as she should be aware of being pregnant and being guided by him in view of the great possibility of her again miscarrying. In about six months I was notified that she was again pregnant, gave her such directions as I considered proper, and in due time I delivered her without instrumental or other artificial aid, of a full grown child, which cried lustily once or twice and seemed vigorous at birth. I tied the cord, detached the infant and laid it in a shawl in a corner of the bed, and, being particularly anxious that my patient should not lose blood unduly, which she could ill afford, placed my left hand over the *fundus* and, finding the uterus well contracted and the placenta detached, removed the latter. While I was just completing this process, I noticed that the infant was very pale and still. It was dead. My first terrible apprehension was that there had been hæmorrhage from the *funis*, but on examination, I found that there had been none. The child had been lying with its face quite uncovered. I told the father that I could not *certainly* explain the cause of death, but that an *autopsy*, if he wonld consent to it, would probably reveal a defect of formation of heart, or, if not that, the cause whatever it might be. He would not consent. He told me that he was quite satisfied that the death was in no way from any fault of mine, and that he would be resigned to it as to an unavoidable misfortune. I attended the mother until my attendance was no longer necessary, met her from time to time, was occasionally called to see her or her husband, and was on the pleasantest terms with both, till, some year and a half after the birth of her ill-starred baby, I met her in the street, saluted her cordially and was "*cut dead.*" I immediately went to her sister-in-law, a very old and attached patient, and asked her what it meant. With a good deal of difficulty I gradually got at the whole story. My patient, who had given me the "cut direct," was led to believe that she was again pregnant, her *menses* not having appeared for over five months. She intended to have me attend her, and followed the directions I had before given her, but a very benevolent and busy neighbor instructed her as to the very superior abilities and "good luck" of a professional brother and urged her, in view of the "bad luck" she had with me, to call in the fortunate relative alluded to. This person called on her, and seeing a woman not far from forty, and being at that time very busily engaged in the study of and effort to obtain reputation in gynæcology, informed the patient that she had uterine disease, and asked her to call at his office. She did so, the next day, the doctor examined her *thoroughly;* you may judge how he examined her from the fact that he informed her that her uterus was very much enlarged, its cavity being over 4½, nearly 5 inches in depth. What he applied or prescribed I know not, nor is it material. The woman went home; about midnight she was seized with intermitting pains and a sanguineous discharge. The *gynæcologist* was sent for, most devotedly remained with his patient for many hours, and did not leave her till he had removed *something*, something which the woman and her husband wished to see, but something which the doctor, good, kind, considerate soul, would not show them. It was "too horrible," "something which Dr. Martin had left in her uterus at the time she had miscarried, over two years before." Now, gentlemen, brethren, of the New York Medical Society, did any of you ever hear of such a case as this so kindly, considerately and disinterestedly explained to poor, simple, credulous folk, by a distinguished brother? Is anything more needful to convince you of the great, the imperative necessity of fully and carefully evacuating the uterus in cases of miscarriage? That a fragment of a fourth month *ovum* should not only have remained in the uterus through and after a pregnancy and parturition at full term, but after that for many consecutive menstrual periods, and in about two years have grown not only too horrible for human sight, but also as large as "a good-sized wash-bowl," is surely very wonderful. Such marvels probably do not happen outside of Roxbury. I sincerely hope that you may be warned by the solemnity of this occurrence and so its repetition in any other part of this highly favored district may be avoided.

Seriously, gentlemen of the society, did you ever hear of a piece of professional ignorance and villainy equal to this I have narrated? A man so ignorant of his profession as to fail to diagnosticate pregnancy in the fifth month, who introduces Simpson's sound, induces abortion, and then, knowing that if he acceded to the natural wish of his poor victim, to see the "tumor" he had so skillfully removed, that no amount of ignorance or credulity could possibly blind her or her husband to the real character of that supposed tumor, refuses to show it, because it is "too horrible," throws on another physician utter blame and odium by an infamous, cowardly, infernal lie; the only way to save himself from the disgrace and ruin he so richly deserved and may yet receive. If our much-lauded Massachusetts Medical Society meant anything—if it was really a means to the end of purifying and elevating the profession of Massachusetts, and not merely a contemptible effete oligarchy, the main end of which is to glorify a *coterie* of mediocrities, and to enable a ring of political doctors to control the profession of the State to its lasting detriment; if it were a society whose chief end was to maintain a proper police and *esprit du corps* in the profession, and not a body of practitioners merely who assemble annually to hear a string of cut and dried and quite impromptu speeches of mutual laudation, listen to a throng of young gentlemen, just back from Vienna, tell them how well they learned their lessons there and see some magical operations done by some Boston gentlemen who are eager to throw all this annual "ground bait" in the fond hope that it may bring fat country cases and fees to their net [the hospital] and their hook [their private practice], this would have been a very proper case to have brought to its notice, but those who know that "venerable" society as I know it, are very well aware that such villainy would meet with but ineffectual and reluctant censure. I long debated, hateful as are such suits, whether it were not best to bring an action at law against this professional "brother," but at last concluded to let it pass, with much more like it, trusting and believing that there will be a final audit of all these things, in a court above human corruption, before a judge with whom perjury and even the influence of the "Boston clique" can avail naught; a court and judge, such as is not to be found even in that very high-minded and quite immaculate body, "THE COUNSELLORS OF THE MASSACHUSETTS MEDICAL SOCIETY."

## Figures and Tables

**Figure f1:**